# Investigation of Amorphous Carbon in Nanostructured Carbon Materials (A Comparative Study by TEM, XPS, Raman Spectroscopy and XRD)

**DOI:** 10.3390/ma16031112

**Published:** 2023-01-27

**Authors:** S. I. Moseenkov, V. L. Kuznetsov, N. A. Zolotarev, B. A. Kolesov, I. P. Prosvirin, A. V. Ishchenko, A. V. Zavorin

**Affiliations:** Boreskov Institute of Catalysis, SB RAS, Lavrentieva 5, Novosibirsk 630090, Russia

**Keywords:** amorphous carbon, nanodiamond, carbon black, multiwalled carbon nanotubes, *sp^2^* carbon, Raman spectroscopy, transmission electron microscopy, X-ray photoelectron spectroscopy

## Abstract

Amorphous carbon (AC) is present in the bulk and on the surface of nanostructured carbon materials (NCMs) and exerts a significant effect on the physical, chemical and mechanical properties of NCMs. Thus, the determination of AC in NCMs is extremely important for controlling the properties of a wide range of materials. In this work, a comparative study of the effect of heat treatment on the structure and content of amorphous carbon in deposited AC film, nanodiamonds, carbon black and multiwalled carbon nanotube samples was carried out by TEM, XPS, XRD and Raman spectroscopy. It has been established that the use of the 7-peak model for fitting the Raman spectra makes it possible not only to isolate the contribution of the modes of amorphous carbon but also to improve the accuracy of fitting the fundamental G and D_2_ (D) modes and obtain a satisfactory convergence between XPS and Raman spectroscopy. The use of this model for fitting the Raman spectra of deposited AC film, ND, CB and MWCNT films demonstrated its validity and effectiveness for investigating the amorphous carbon in various carbon systems and its applicability in comparative studies of other NCMs.

## 1. Introduction

Nanostructured carbon materials (NCMs)—carbon black (CB), carbon fibers (CFs), multiwalled carbon nanotubes (MWNTs), nanodiamonds (NDs) and onion-like carbon (OLC)—are among the most promising materials in various practical applications due to their unique electrophysical and chemical properties. The application field of NCMs is extremely wide: fillers in structural materials (polymer, ceramic and carbon-carbon composites) for the automotive, aviation and space industries; structuring additives in the anode and cathode materials for electrochemical energy sources; catalyst supports and sorbents in chemical processes and medicine; sensitive elements of sensors and much more [1,2,3,4,5]. NCMs are commonly obtained at high temperatures of 600 °C and above. Due to this, amorphous carbon is present in the bulk and on the surface of NCMs. Amorphous carbon has a significant effect on both the mechanical properties of NCMs (strength, elasticity, modulus of elasticity, etc.) and their physicochemical properties (the interaction of NCM with a matrix in composites, substrate in supports and catalysts, corrosion resistance in electrochemical applications and thermal and electrical conductivity of contacts between NCM particles in composites) [6,7,8,9,10]. Thus, the determination of AC in NCMs is extremely important for controlling the properties of a wide range of materials, from construction materials to sensors and catalysts.

To determine the content of amorphous carbon in samples, a wide range of methods were used: transmission electron microscopy (TEM)—direct observation of graphene fragments in the sample structure; X-ray diffraction analysis (XRD)—analysis of the background on the diffraction pattern in the region of the (002) reflection [11,12,13], where the proportion of amorphous carbon is defined as the ratio of the area bounded by the baseline of the peak and the *x*-axis to the total area under the peak. Another way to find the content of amorphous carbon is to estimate the ratio of reflection intensities I(112)/I(110) [14]. The use of X-ray photoelectron spectroscopy (XPS) makes it possible to separate the states of carbon atoms (*sp^1^-*, *sp^2^-*, *sp^3^*-hybridization, various C-C and C-O bonds) in the surface layer of the sample by analyzing the binding energy of photoelectrons and using these data to calculate the fraction of amorphous carbon in the surface layer. Another way to observe the structure of all types of carbon materials is through Raman spectroscopy. Graphite, diamond, graphene, nanotubes, fullerenes in the form of single crystals, polycrystalline films and powders exhibit their own characteristics in the Raman spectra and therefore can be identified by this method [15,16,17,18,19,20,21,22,23]. Graphene fragments can be considered the building blocks of *sp^2^* carbon materials, the size of which is one of the most important characteristics of the ordering of carbon materials. The Raman spectra of NCM in the single-phonon scattering region contain two broad and strongly overlapping peaks that correspond to the D and G lines. Mode G (1578–1587 cm^–1^) is characteristic of ordered graphitized systems and is associated with planar asymmetric stretching vibrations of C=C bonds with the E_2g_ symmetry [24,25]. Mode D (1340–1348 cm^–1^) is usually associated with the violation of the selection rules due to the finite sizes of crystallites, disorder and various kinds of defects [26,27]. In the region of two-phonon scattering, NCM is characterized by the presence of a 2D mode, which indicates the number of graphene layers and their mutual orientation (superposition order) [18,19,22]. As a rule, scattering in the region of two-phonon interaction is not typical of small graphene layers, for example, in amorphous carbon, which is close in size to polyaromatic molecules.

In [28], the authors carried out a deeper analysis of the obtained Raman spectra for carbon black, spark discharge soot, etc., and found that for NCMs in the region of single-phonon scattering, several characteristic bands can be additionally distinguished. It was found that D_2_ (D’) mode (~1620 cm^–1^) corresponds to disordered graphitic lattice (surface graphene layers, E_2g_ symmetry) [29], D_3_ mode (D’’, A, ~1500 cm^–1^) corresponds to amorphous carbon (Gaussian [30] or Lorentzian [31,32,33] line shape) and D_4_ mode (I, ~1200 cm^–1^) is a disordered graphitic lattice (A_1g_ symmetry) [34], polyenes [31,33] and ionic impurities [32].

Using this approach to fitting the Raman spectra, the authors of [35,36] analyzed amorphous carbon contained in sedimentary rocks that formed at different temperatures (165–655 °C). It has been shown that with an increase in the temperature of sedimentary rock formation, a transformation of the Raman spectra of carbon is observed: the full width at half maximum (FWHM) of the D_1_ and D_2_ bands decreases linearly with increasing metamorphic temperature in the range of approximately 150–400 °C. It is shown that an increase in the temperature of sedimentary rock formations in the range of 150–400 °C leads to a linear decrease in the FWHM of the D_1_ and D_2_ modes of the carbon Raman spectra. It was also found that, depending on the formation temperature of sedimentary rocks, different models were required for an adequate approximation of the Raman spectra. Thus, the complexity of the model used by the authors increased as the sample formation temperature decreased from peak 1 (655 °C) to peak 5 (301 °C).

In paper [37], the Raman spectra of carbon fibers were studied. In this work, models with different numbers of peaks (2–8) were evaluated for fitting the Raman spectra of standard modulus (SM) and intermediate modulus (IM) carbon fibers, and 2- and 3-peak models were shown to be insufficient for such materials. The 4-peak model is probably sufficient to identify general qualitative trends, but the 5-peak fitting model consisting of D_1_, D_2_, D_3_, D_4_ and G peaks should be used to establish any reliable relationships between spectral parameters and physical or mechanical parameters. High modulus (HM) analysis of carbon fibers indicates the presence of at least six peaks corresponding to D_1_, D_2_, D_3_, D’’, D_4_ and G modes. The authors note that the introduction of additional peaks into the model improves the accuracy of the fitting, but there is no physical motivation for this.

To understand the physical motivation for introducing additional peaks into the Raman fitting model, it is expedient to analyze the dependences of the Raman spectra of various nanosized carbon fragments (see Sheka et al. [38,39,40]). In the works, the Raman spectra were modeled for a large number of polyaromatic fragments and graphenes of various structures, as well as the structures that can be associated with amorphous carbon present in NCM. It is shown that the practical identification of carbon fragments by their Raman spectra causes significant difficulties, since the Raman spectra are determined not only by the structure of the fragment itself but also by its environment. It should be recognized that the presence of broad bands in the spectra of amorphous carbon is due to an extremely large set of nanosized fragments of various structures.

The use of high resolution (HR) TEM has significant limitations in studying the structure of amorphous forms of carbon due to its possible graphitization under the action of an electron beam [41,42,43]. At the same time, the analysis of amorphous forms of carbon makes it possible to fix the formation of ordered fragments of graphene starting from ~1 nm, which corresponds to fragments containing 5–7 benzene rings [44]. For more ordered carbon systems, TEM can reveal the mutual arrangement of *sp^2^* fragments (*sp^3^* for ND) and characterize the presence of structural defects in them.

In view of the foregoing, the main objective of this work was to study the relationship between the structure of amorphous carbon and its Raman spectra, to develop a physical motivation for using a multipeak model of fitting Raman spectra to expand the possibilities of Raman spectroscopy for the identification of amorphous carbon, and to test this approach when studying a wide range of NCMs.

## 2. Materials and Methods

### 2.1. Nanostructured Carbon Materials (NCMs)

**Amorphous carbon (AC)** was obtained on a crystalline silicon substrate by vacuum deposition (VUP-4, Kazan, Russia). The pressure in the chamber during the deposition was 10^–5^ mbar; the evaporator current was ~200 A; and the deposition time was ~10 s. Heat treatment of amorphous carbon for Raman spectroscopy was carried out at a pressure of 10^–7^ mbar and temperatures of 30, 150, 300, 450 and 600 °C for 1 h.

We used the **detonation nanodiamond (ND)** afforded by FGUP “Altai” (Russia) [45]. The size of primary ND particles was 3–5 nm, and the size of primary agglomerates was 100–150 nm. The remaining non-*sp^3^* carbon was removed by additional purification of ND by boiling in a mixture of concentrated perchloric and sulfuric acids (1:1) for 2 h, which was followed by washing with distilled water until neutral pH and drying in air at 80 °C for 3 days. Heat treatment of ND was carried out in a quartz ampoule at a pressure of 10^–2^ mbar (an additional trap with liquid nitrogen was used) and temperatures of 650, 750, 850 and 950 °C for 1 h.

A Printex XE2-B sample (Orion Engineering Carbons, Germany) was used as **carbon black (CB)**. Its production temperature is approximately 800 °C.

**MWCNTs** with a mean diameter of 9.8 nm were produced using the CVD method via ethylene decomposition with a bimetallic Fe-Co/Al_2_O_3_ catalyst at 680 °C [46,47]. This catalyst, with the active component Fe_2_Co, has been shown to produce MWCNTs of low defect severity and with a low content of inorganic impurities. Subsequent refluxing of nanotubes with 15% HCl (followed by washing with distilled water until neutral pH and subsequent drying in air) allows decreasing the content of catalyst traces to 0.2–0.3 wt%.

**Preparation of heat-treated CB and MWCNTs**. The samples were heated at a pressure of 10^–6^ mbar by the electron impact method. The sample was placed in a tantalum cuvette measuring12 × 12 mm in size and 2 mm in depth. A tungsten wire spiral 0.15, mm in diameter, was placed under the cuvette at a distance of 4 mm. A current of 4–5 A was passed through the spiral so that its temperature was not lower than 2200 °C. A voltage of 1000–1500 V was applied to the cuvette (anode) and the tungsten spiral (cathode). The sample temperature was controlled by changing the applied voltage. The temperature of the spiral and sample was controlled using an optical reference pyrometer, taking into account the emissivity of tantalum (0.25) and tungsten (0.32). The high-temperature treatment time for each sample was 5 min.

### 2.2. Methods

The structure of catalysts and MWCNTs was also characterized with **transmission electron microscopy** (**TEM**, Themis-Z 3.1 (TFS, USA), and JEM 2010 Jeol (Japan)). For TEM characterization of the specimen structure, a sample was deposited on a copper grid with a carbon film. TEM images were processed using the ImageJ program [48].

**XPS measurements** were performed inside a photoelectron spectrometer (SPECS, Germany) equipped with four chambers, i.e., the load lock, preparation, analyzer, and high-pressure cell (HCP) chambers. The analyzer chamber is equipped with a hemispherical analyzer PHOIBOS-150-MCD-9, an ellipsoidal monochromator FOCUS 500 and an X-ray source XR 50 M with a double Al/Ag anode. In the present work, AlK_α_ (hν = 1486.74 eV, 200 W) was used as a primary radiation source. The binding energy (BE) scale was precalibrated using the positions of Au4f_7/2_ (84.0 eV) and Cu2p_3/2_ (932.7 eV) core-level lines from metallic gold and copper foils. Spectral analysis and data processing were performed with XPS Peak 4.1 software. The position of C1s line at BE = 284.5 eV from the support (HOPG) was used as an internal standard for the calibration of other measured peaks [49]. The pressure in the analyzer chamber did not exceed 5 × 10^−9^ mbar. The curve-fitting procedure was carried out using approximation by a combination of the Gaussian and Lorentzian or Doniach-Sunjic functions with subtraction of the Shirley-type background [50,51]. Before the curve fitting, all experimental spectra were smoothed using a Fourier filter. No considerable difference between the smoothed and experimental curves was observed (the mean-square deviation was less than 1%). Before recording the XPS spectra, the sample was kept at 150, 300, 450 and 600 °C for 1 h in the vacuum chamber of the spectrometer. The C1s and O1s detailed core-level spectra were measured after each treatment. For the quantitative analysis, the integral intensities of the measured peaks in XPS the spectra (C1s core levels) were corrected to their respective atomic sensitivity factors.

**XRD** patterns of the samples were obtained on an ARL XTRA X-ray diffractometer (Switzerland) with CuK_α_ radiation in the angle range 15–85° (2θ) at a 0.02° step and 4 °/min rate. A Mythen2 R1D detector (Dektris, Switzerland) was employed.

**Raman spectra characterization** of the NCMs was performed on a LabRAM Horiba (Japan) single-stage spectrometer with a CCD Symphony detector (Jobin Yvon, France) having 2048 horizontal pixels. A laser’s power (the 514 nm line of an Ar^+^ laser) on the sample was typically less than 0.1 mW. A spectral resolution was 3.0 cm^–1^. Single spectra were collected from the regions with a cross section of approximately 1 μm. From three to five spectra were obtained for each sample. The spectra were processed using the Fityk software [52]. The initial Raman spectra were smoothed (moving average over three points), the shape of the peaks was set as Voigt and the fitting method was Levenberg–Marquardt (MPFIT library).

## 3. Results and Discussion

### 3.1. Amorphous Carbon

A study on the transformation of the amorphous carbon structure and properties during heat treatment was carried out via TEM, XPS and Raman spectroscopy.

#### 3.1.1. TEM of Heat-Treated Amorphous Carbon

**Figure 1:**
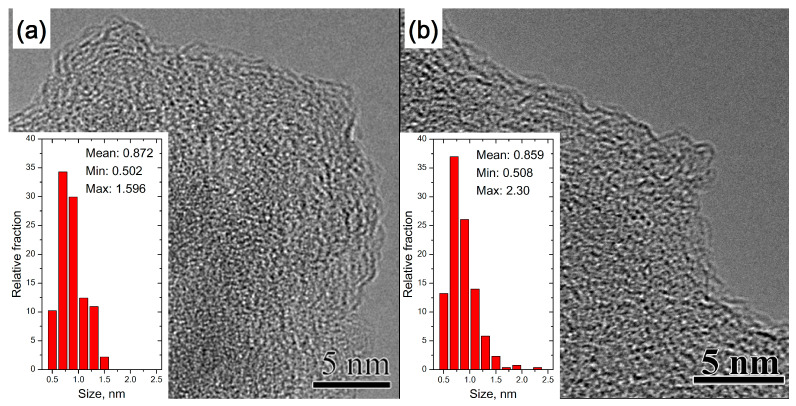
Typical TEM images of fragments of AC samples used to determine the average length of carbon *sp^2^* fragments on the AC layer surface. (**a**) The initial AC sample was treated at a temperature of 30 °C, and (**b**) the AC sample was treated at 600 °C.

Figure 1 shows typical TEM images of AC films before (30 °C) and after (600 °C) heat treatment. It has been established that heat treatment at 600 °C does not lead to a noticeable change in the structure of amorphous carbon. The analysis of the average length of *sp^2^* carbon fragments of AC from TEM images showed that in both cases it was 0.86–0.87 nm. At the same time, a large number of *sp^2^* fragments 0.6–1 nm in size, due to the large statistical weight, makes it difficult to establish the fact of an increase in the size of *sp^2^* fragments by calculating the average size. However, the TEM images of the heated AC contain more elongated *sp^2^* carbon fragments up to 2.3 nm in length compared to 1.6 nm for the initial AC, which may be a consequence of AC ordering during heat treatment at 600 °C.

#### 3.1.2. XPS of Heat-Treated Amorphous Carbon

**Figure 2:**
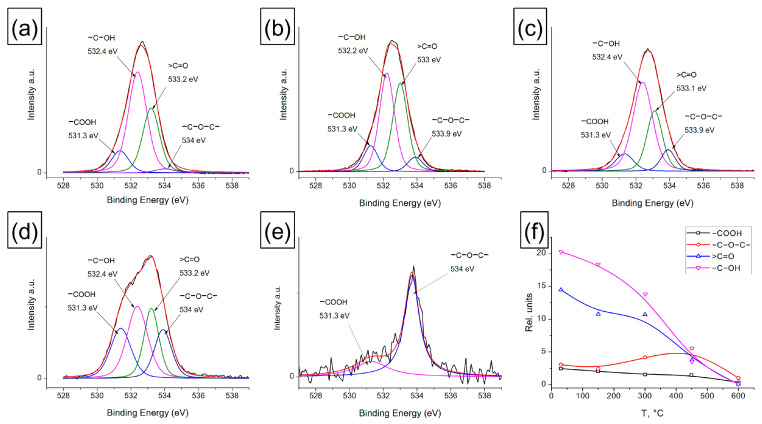
XPS decomposition of the O1s spectra of the AC film heated at different temperatures: (**a**) initial sample, 30 °C; (**b**) 150 °C; (**c**) 300 °C; (**d**) 450 °C; (**e**) 600 °C; and (**f**) changes in the content of oxygen-containing functional groups on the AC surface depending on the heating temperature.

**Figure 3:**
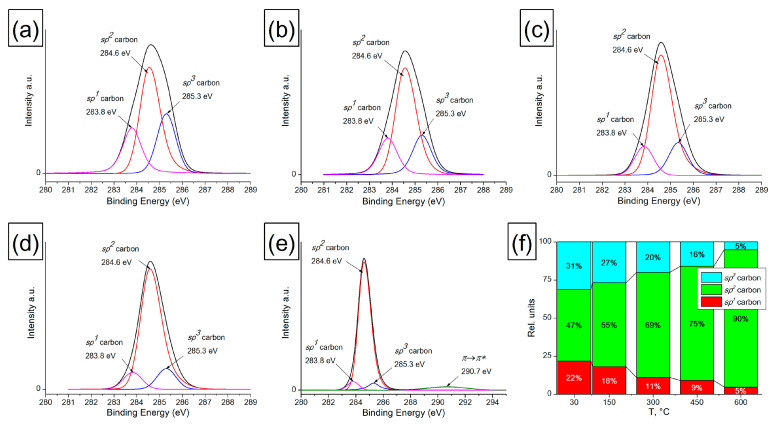
XPS decomposition of the C1s spectra of the AC film heated at different temperatures: (**a**) initial sample, 30 °C; (**b**) 150 °C; (**c**) 300 °C; (**d**) 450 °C; (**e**) 600 °C; and (**f**) changes in the proportion of *sp^1^*, *sp^2^* and *sp^3^* hybridized carbon atoms on the AC surface depending on the heating temperature.

The change in the state of near-surface carbon atoms in AC films was also studied by XPS. Figure 2 and Figure 3 present the XPS decompositions of the O1s and C1s spectra for an AC film treated at 150, 300, 450 and 600 °C. When processing the O1s spectra using XPS, the FWHM values (1.2 eV) and the positions of each of the peaks were fixed. To decompose the O1s peak, four components corresponding to the following oxygen-containing surface groups were used: carboxyl group −COOH (531.3 eV), carbonyl group >C=O (533.1 eV), hydroxyl group -C-OH (532.4 eV) and ether group -C-O-C- (533.8 eV). The data obtained on the integral intensities, taking into account the sensitivity coefficient (for C, it is 1, for O, it is—2.93) and the stoichiometry of oxygen and carbon in the corresponding oxygen-containing groups, were used in processing the C1s spectra and determining the proportion of *sp^1^*, *sp^2^* and *sp^3^* hybridized carbon atoms. For this, all oxygen-containing groups were subtracted from the C1s spectrum. The decomposition of the resulting C1s spectrum is shown in Figure 3. After XPS processing of the C1s spectra, the following values were fixed: FWHM (0.97 eV) and the position of each of the peaks (283.8–284.1 eV corresponds to carbon *sp^1^*, 284.6 eV—to carbon *sp^2^* and 285.2–285.4 eV—to carbon *sp^3^* on the AC surface). In addition, when conductivity appears in the film, the π→π* peak at 290.7 eV is observed, which has an asymmetric shape with the given symmetry coefficients and belongs to the *sp^2^* form of carbon [50].

It has been established that oxygen-containing groups are removed from the AC surface during heat treatment. Up to a temperature of 450 °C, most of the carbonyl and hydroxyl groups are destroyed, and at a heating temperature of 600 °C, mainly ester groups -C-O-C- (1%) and a small part of −COOH groups (0.35%) remain on the surface. In the temperature range of 150–450 °C, the content of ester groups on the AC surface increases, which may be due to the rearrangement of carbonyl and hydroxyl groups accompanied by CO evolution and the formation of ester groups. It is known that the presence of oxygen-containing groups in carbon materials makes it possible to stabilize their surfaces.

The removal of the functional group leads to spontaneous graphitization of the surface with the closure of dangling C-bonds and the formation of *sp^2^*-hybridized carbon [53]. In contrast to nanodiamonds, in the case of amorphous carbon, an increase in the fraction of *sp^2^*-hybridized carbon is determined over the entire range of heating temperatures. This is confirmed by the data on the hybridization state of C atoms obtained from the analysis of the C1s peak, where a decrease in the fraction of *sp^1^* and *sp^3^* is observed and a monotonic increase in the fraction of carbon *sp^2^* with increasing heating temperature is observed uniformly over the entire temperature range of 30–600 °C. Additionally, the appearance of the π→π* 290.7 eV peak in the spectra of the sample treated at 600 °C indicates the beginning of AC graphitization and the formation of a conducting system based on *sp^2^* carbon.

#### 3.1.3. Raman of Heat-Treated Amorphous Carbon

**Figure 4:**
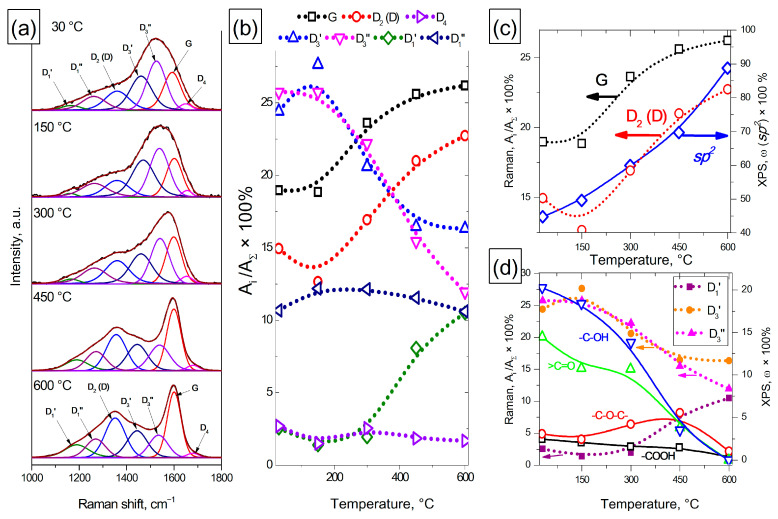
Typical Raman spectra of AC samples treated at different temperatures (**a**). A change in the area of the peaks of the 7-peak model depends on the temperature of the AC treatment (**b**). Changes in the fraction of G and D_2_ (D) modes and changes in the fraction of carbon *sp^2^* (by XPS) on the AC surface depend on the treatment temperature (**c**). Changes in the fraction of D_1_′, D_3_′ and D_3_″ modes and changes in the proportion of oxygen-containing groups (by XPS) on the AC surface depending on the treatment temperature (**d**).

The Raman study of AC samples showed that in the region of one-phonon scattering (1000–1800 cm^–1^), there were significant changes in the spectra with increasing treatment temperature (Figure 4a).

We used a 7-peak model to fit the obtained Raman spectra. Since in our experiments the intensity of the bands and their ratio of intensities varied over a wide range, the numbering was carried out in the ascending Raman shift so as not to confuse the reader. Mode D_1_′ (1110–1190 cm^–1^) corresponds to a combination of vibrations caused by the chain stretching containing vinyl groups, C-H wagging modes, heteroatoms and *sp^2^* carbon atoms located in defects and in an amorphous phase, polyene and polyyne fragments in the AC structure [54]; mode D_1_″ (1210–1290 cm^–1^) is included in the model because it was present in the Raman spectra of carbon black and MWCNTs treated at 2000–2600 °C and in the spectra of NDs treated at 650–950 °C, as well as for carbon fibers SM and IM in the article [37]. In [55], the authors suggest that the presence of this mode might be rather related to the nature of defects that are present or not in each type of carbon, including point defects, edge planes, stacking faults, curved planes, twisted planes or others. The peak at 1310–1390 cm^–1^ (D_2_ (D) mode) is due to a vibration with the A_1g_ symmetry, which is not present in perfect graphite and exists only in disordered carbon structures. The selection of the vibration modes is broken due to a disorder and defects existing in the graphitic lattice (an increase in the ratio of edge planes/basal planes, a decrease in the size of the crystallites, grain boundaries, amorphous carbon, and doping). Modes D_3_′ (1450–1490 cm^–1^) and D_3_″ (1520–1555 cm^–1^) are associated with the presence of amorphous carbon [33], a peak located at 1575–1600 cm^–1^ (G mode) is due to graphitic in-plane vibrations with the E_2g_ symmetry involving an in-plane bond-stretching motion of C *sp^2^* atom pairs; and D_4_ mode (1615–1650 cm^–1^) is related to lattice vibrations similar to G, but includes vibrations from graphene layers on the surface of a graphite-like crystal [34]. The position of the peak maxima was limited to the above ranges, and R^2^ was at least 0.995 for all the spectra when fitted.

It can be seen that in the Raman spectra of the initial and treated at 150 °C samples of amorphous carbon, the characteristic modes of amorphous carbon (D_3_′ and D_3_″) dominate, and the other modes do not change significantly (Figure 4b). It is also necessary to note an increase in the fraction of D_3_′ mode during heat treatment at 150 °C. A further increase in the treatment temperature in the range of 150–600 °C leads to an increase in the fraction of G mode, which indicates an increase in the size of non-defective graphene fragments in the AC structure. At the same time, a decrease in the fraction of amorphous carbon modes D_3_′ and D_3_″ is observed. In the temperature range of 300–600 °C, an increase in the fraction of D_1_′ mode is observed and the remaining modes (D_1_″ and D_4_) do not change significantly during heat treatment. It should be noted that D_3_′ and D_3_″ modes seem to characterize different types of amorphous carbon, since they have different dependences on the AC sample treatment temperature.

A joint analysis of the data obtained by TEM, XPS and Raman revealed a correlation between them. Thus, according to the XPS data, the fraction of *sp^2^* carbon in the AC samples increases over the entire AC treatment range of 30–600 °C, while the fractions of G and D_2_ (D) modes in the AC samples also increase at temperatures in the range of 150–600 °C (Figure 4c). At the same time, in the temperature range of 30–150 °C, an increase in the fraction of D_3_′ amorphous carbon mode is observed, while the fraction of other modes does not change significantly. This allows us to conclude that the destruction of hydroxyl (-C-OH) and carbonyl (>C=O) groups leads to additional amorphization of the AC surface. An increase in the treatment temperature in the range of 150–600 °C is accompanied by further destruction of functional groups on the AC surface and leads to a decrease in the fraction of amorphous carbon modes and a simultaneous increase in the fraction of G and D_2_ (D) modes. An increase in the fraction of D_1_′ mode in the range of 300–600 °C indicates the formation of such defects as *sp^2^* carbon atoms located in defects and in an amorphous phase. The results obtained are also confirmed by a TEM study on the structure of amorphous carbon, which demonstrates an increase in the maximum length of *sp^2^* fragments in the AC structure from 1.6 to 2.3 nm with an increase in the heat treatment temperature to 600 °C.

Thus, joint studies of changes in the structure of amorphous carbon under the action of heat treatment allow us to conclude that it is possible to use the method of Raman spectroscopy and the 7-peak model of fitting the Raman spectra for a qualitative analysis of changes in the structure of highly defective carbon materials, such as amorphous carbon, and, in particular, the content of amorphous carbon in the samples and the change in its fraction during heat treatment. The use of the 7-peak model made it possible to distinguish two types of amorphous carbon characterized by different modes in the Raman spectra. In addition, the selection in this model of the contribution of the D_3_′ and D_3_″ amorphous carbon peaks, as well as the D_1_′ and D_1_″ modes, allows increasing the accuracy of fitting the main peaks of G and D_2_ (D) modes, and obtaining a satisfactory convergence between XPS data and Raman spectroscopy.

### 3.2. Nanodiamond

Nanodiamonds heated at temperatures of 650, 750, 850 and 950 °C were studied to assess the possibility of practical use of the proposed approach for applying the 7-peak model to estimate changes in the fraction of amorphous carbon with increasing temperatures of ND heat treatment. The graphitization of the ND surface was previously studied in detail by us in [53,56,57]. It has been established that surface graphitization begins with the removal of surface groups that stabilize the surface carbon atoms of ND. When such groups are removed, the formation of nanosized *sp^2^* fragments begins. The size of the *sp^2^* fragments is comparable to the size of the faces of diamond nanoparticles (<5–7 nm).

#### 3.2.1. TEM Characterization of ND

**Figure 5:**
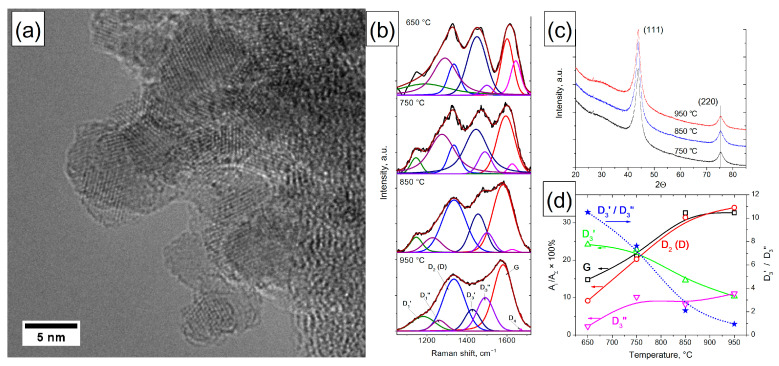
Typical TEM image of ND (**a**). Changes in the Raman spectra of ND during heat treatment and their approximation using the 7-peak model (**b**). XRD data for ND (**c**). Changes in the fraction of G, D_2_ (D) and D_3_′ and D_3_″ modes in the Raman spectra of ND heated at different temperatures; changes in the D_3_′/D_3_″ ratio with temperature (**d**).

It has been established that NDs are the primary agglomerates, 100–150 nm in size, consisting of the primary ND particles with a size of 3–5 nm. Graphene-like structures can be seen on the surface of primary particles (Figure 5a). However, it is not possible to determine their size and quantity in ND samples by this method due to ND graphitization under the action of the microscope electron beam (even at accelerating voltages of 60–70 kV).

#### 3.2.2. XRD Characterization of ND

It was found that ND is characterized by the presence of two reflections, (111) and (220), which do not change when the treatment temperature is varied in the range of 750–950 °C (Figure 5c). Small variations in the diffraction pattern in the range of 2θ angles 24.5–35.5 may be caused by the onset of ND surface restructuring during graphitization [56] and by the difference in sample packing.

#### 3.2.3. Raman Characterization of ND

Raman characterization of ND showed that dramatic changes were observed in the spectra with an increase in the ND treatment temperature in the range of 650–950 °C (Figure 5b). Thus, in the spectra of ND with a treatment temperature of 650–750 °C, three to four broad peaks are observed; then the number of distinct peaks decreases to two with an increase in the treatment temperature to 950 °C. The use of the 7-peak fitting model revealed that D_1_′ and D_1_″ modes make a large contribution—up to 15% of the region—to the full spectrum. The presence of these modes is due to the presence of *sp^2^* carbon atoms located in defects and in the amorphous phase, including point defects, edge planes, stacking faults, curved planes and twisted planes, which is consistent with the model of ND graphitization during heat treatment [56].

The use of the 7-band model also allowed us to evaluate the change in the amorphous and graphitized carbon components in ND depending on the treatment temperature. It was found that the fraction of G and D_2_ (D) modes increases with an increase in the treatment temperature from 650 to 850 °C and does not change significantly in the range of 850–950 °C (Figure 5d). The change in the fraction of D_3_′ and D_3_″ modes is multidirectional: the fraction of D_3_′ mode monotonically decreases with increasing treatment temperature, and the fraction of D_3_″ mode increases in the temperature range of 650–750 °C and then changes slightly. In this case, the ratio of the areas of D_3_′ and D_3_″ peaks decreases from 10.5 to 0.9 with increasing temperature. It is known from our previous work that the full decomposition of oxygen-containing groups occurring at 900 °C of ND results in its partial surface graphitization (by XPS and temperature-programed desorption (TPD)). The onset of ND graphitization occurs at 950 °C, which is supported by the appearance of the *sp^2^* component in the C1s spectrum [53,56,57]. Thus, during the destruction of the main amount of ND surface groups (650–850 °C), the accumulation of highly defective *sp^2^* carbon fragments on the ND surface is observed. These fragments combine into highly defective *sp^2^* graphene-like ND shells up to a temperature of 850 °C (increasing the G and D_2_ (D) modes). At a temperature of 850–950 °C, the formation of such shells is basically complete, which is expressed in a slight change in the fraction of G and D_2_ (D) modes in this temperature range. Amorphous carbon also participates in the formation of highly defective *sp^2^* graphene-like ND shells, which is confirmed by a decrease in the fraction of the D_3_′ mode.

The results obtained are consistent with the optical properties of ND heated up to 950 °C [58]. It was found that an increase in the annealing temperature of ND leads to a systematic decrease in reflectance in the range of 200–900 nm, accompanied by a shift of the absorption maximum to the low-frequency spectrum range. The absorption maximum of ND annealed at 300–850 °C shows a significant bathochromic shift from 250 nm, which is characteristic of the pristine sample, up to 538 nm and an increase in the absorption maximum from 88.6 to 94.9%. A further increase in the treatment temperature in the range of 850–950 °C leads to a shift in the absorption maximum to 635 nm, while the absorption increases only from 94.9 to 95.5%. This observation can be explained in terms of the systematic growth of the size of graphene flakes up to a size comparable with the ND crystal planes of ND primary particles (1–5 nm). This correlates with the change in the contribution of G and D_2_ (D) modes in the Raman spectra of ND, where, up to a temperature of 850 °C, the size of *sp^2^* fragments increases to the size of primary ND particles, which coincides with an increase in the contribution of G and D_2_ (D) peaks, and further up to 950 °C, the contribution of G and D_2_ (D) modes changes insignificantly. The absorbance spectra of condensed aromatic compounds with an increasing number of aromatic rings are characterized by a bathochromic shift (because of the p band’s optical transition). However, optical absorbance is growing in a wide region because of the coexistence of graphene flakes of very different sizes in the sample. The change in the fraction of D_3_′ and D_3_″ modes of amorphous carbon also correlates with the absorption data in the optical range. In the temperature range of 850–950 °C, when the ratio of areas of the Raman peaks D_3_′/D_3_″ is less than 2–3, there is a broadening of the absorption peaks in the optical range. This allows us to conclude that D_3_′ mode is determined mainly by small *sp^2^* graphene-like carbon fragments (since the bathochromic shift is small), while D_3_″ mode is determined by larger *sp^2^* graphene-like carbon fragments. Modeling of the electronic spectra of planar graphene fragments of *sp^2^* carbon (decorated with H) in the HyperChem 8.0 program (ZINDO/S) showed that the absorption maximum of 500–540 nm corresponds to a graphene-like fragment size of 1.5–1.7 nm. Thus, this suggests that the D_3_′ mode in the Raman spectra of NCMs is due to the presence of graphene-like fragments up to 1.5–1.7 nm in size, and the D_3_″ mode is more than 1.7 nm.

Consequently, the established temperature dependences of the ratios of G and D_2_ (D) modes, as well as D_3_′ and D_3_″ in the Raman spectra of ND treated at 650–950 °C, agree with the data obtained by XPS, electron reflection spectroscopy, temperature-programed desorption and the graphitization model for ND. A comparison of experimental and model data made it possible to estimate the critical size of a graphene-like *sp^2^* fragment at a level of 1.5–1.7 nm; below it, it contributes to D_3_′ mode, and above it, to D_3_″ mode.

### 3.3. Carbon Black Printex-XE2-B

#### 3.3.1. XRD Characterization of the Structure of Initial CB

**Figure 6:**
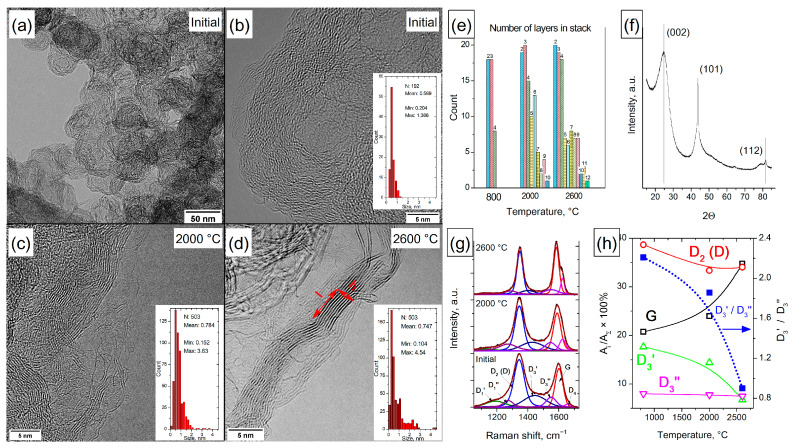
Typical TEM images of carbon black Printex-XE2-B treated at different temperatures: initial (800 °C—(**a**,**b**)), annealed at 2000 °C (**c**) and annealed at 2600 °C (**d**). The insets show the length distribution of *sp^2^* fragments on the particle surface. Dependence of the thickness of the stacks of structured *sp^2^* carbon in Printex-XE2-B on the treatment temperature (**e**). XRD of the initial Printex-XE2-B (**f**). Typical Raman spectra of Printex-XE2-B before and after heat treatment (**g**). Changes in the fraction of G, D_2_ (D), and D_3_′ and D_3_″ modes in the Raman spectra of Printex-XE2-B during heat treatment; and changes in the D_3_′/D_3_″ ratio with temperature (**h**).

The XRD characterization of the structure of the initial CB is shown in Figure 6f. It was found that the diffraction pattern contains reflections 2θ 24.7, 43.7 and 81.4°, corresponding to the hkl (002), (101) and (112) faces and interplanar distances of 0.359, 0.206 and 0.117 nm, respectively. For these reflections, the CSRs were 2.19, 18.15 and 10.59 nm.

#### 3.3.2. TEM Comparative Study

The TEM comparative study of the initial sample and that treated at 2000–2600 °C is illustrated in Figure 6a. It can be seen that the CB has a globular structure; the globules are 50–100 nm in size and are consolidated into aggregates by outer shells. The globules of the initial sample (at the synthesis temperature of ~800 °C) consist of highly defective graphene layers with a total thickness of 4–7 nm. It was found that high-temperature treatment leads to ordering and increases the length of defect-free graphene layers that form the walls of the globules. Thus, before heat treatment, the mean length of graphene fragments was approximately 0.57 nm and after heat treatment at a temperature of 2000–2600 °C, the mean size of defect-free areas reached 0.75–0.78 nm. Analysis of the TEM image of carbon black after heat treatment showed an increase in the number of graphene layers in ordered stacks (Figure 6e). The maximum number of graphene fragments in a stack for the initial CB did not exceed 4; for those treated at 2000 °C, 10 fragments; and for 2600 °C, 12. In addition, it was found that the maximum length of graphene fragments varied from 1.3 nm for the original CB (800 °C) to 3.6 nm (2000 °C) and 4.5 nm for CB treated at 2600 °C. The data obtained indicated the transformation of disordered *sp^2^* fragments into graphite-like carbon during heat treatment.

#### 3.3.3. Raman Spectroscopy Characterization

Raman spectroscopy characterization of the initial and treated CB Printex-XE2-B samples and its fitting using the 7-peak model are shown in Figure 6g. It has been established that with an increase in the treatment temperature, the shoulder below 1250 cm^–1^ decreases. It corresponds to D_1_′ and D_1_″ modes due to the presence of *sp^2^* carbon atoms located in defects and in the amorphous phase, point defects, edge planes, stacking faults and curved planes. The decrease in intensity of the spectrum in the region of 1400–1550 cm^–1^, which corresponds to amorphous carbon (modes D_3_′ and D_3_″), occurs mainly due to a decrease in the fraction of D_3_′ mode corresponding to graphene fragments smaller than 1.7 nm; the D_3_′/D_3_″ ratio decreases from 2.2 for the initial CB to 0.9 for that heated at 2600 °C. An increase in the fraction of G and D_4_ modes is also observed (Figure 6g,h). The observed changes are associated with the ordering of the CB structure, and a higher heating temperature corresponds to a more ideal structure with fewer defects in the graphene layer. Raman spectroscopy data were used to evaluate changes in the size of graphitized blocks in Printex-XE2-B samples (Table 1). This was made using the models developed by Tuinstra and Koenig [59] as well as Cançado [20].

A comparison of the results obtained by the analysis of TEM images of CBs with the results of processing the Raman spectra showed that the estimation of the length of graphene fragments by the Tuinstra and Koenig method is more suitable for determining their length in the case of CB.

It should be noted that the treatment of CB at 2000 °C leads to a decrease in the fraction of D_2_ (D) mode from 39 to 33%, and a further increase in the heating temperature to 2600 °C has virtually no effect on the fraction of D_2_ (D) mode. At the same time, the contribution of the D_3_″ mode, even at a treatment temperature of 2600 °C, is ~7%, which indicates that highly defective *sp^2^* carbon fragments are retained in the sample structure. The reason for this may be that CB Printex-XE2-B belongs to the class of difficult-to-graphitize carbon blacks [60], whose structure is represented by randomly oriented, well-graphitized *sp^2^* carbon blocks interconnected to each other by several graphene layers. The presence of such layers prevents both the stacking of graphitized blocks during heat treatment and the complete carbon black graphitization.

Thus, it has been established that the use of the 7-peak fitting model for the analysis of the Raman spectra of CBs makes it possible to reveal the presence of amorphous and weakly ordered *sp^2^* carbons in their structure and separate them over a wide range of heat treatment temperatures. The results obtained were confirmed by the TEM method and data on the structure of difficult-to-graphitize soot.

### 3.4. MWCNTs

#### 3.4.1. TEM Characterization

**Figure 7:**
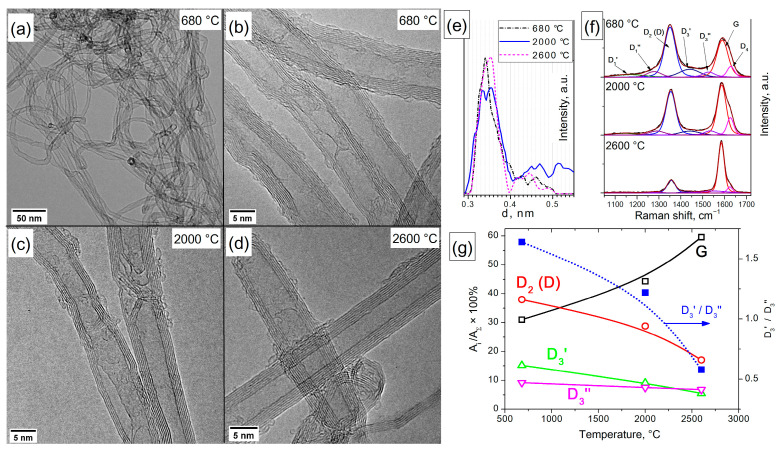
Typical TEM images of MWCNTs treated at different temperatures: low resolution, initial nanotubes (**a**); high resolution, initial nanotubes (**b**); high resolution, heated at 2000 °C (**c**); and high resolution, heated at 2600 °C (**d**). Estimation of the distribution of interlayer distances of MWCNTs depending on the treatment temperature (**e**). Typical Raman spectra of MWCNTs before and after heat treatment (**f**). Changes in the fraction of G, D_2_ (D), D_3_′ and D_3_″ modes in the Raman spectra of MWCNTs during heat treatment; changes in the D_3_′/D_3_″ ratio with temperature (**g**).

The TEM characterization of the structure of the initial and heat-treated MWCNTs is illustrated in Figure 7a–d. It has been established that individual nanotubes intertwine and form “lumps”. Thermal treatment of MWCNTs at temperatures of 2000 and 2600 °C leads to the formation of a more ideal structure. Treatment at 2000 °C leads to a decrease in the number of single-carbon *sp^2^* fragments on the surface of nanotubes, an equalization of the number of graphene layers over the cross-section of nanotubes and the disappearance of such defects as dangling layers and Y-junctions to the walls. When nanotubes are treated at 2600 °C, their shape additionally changes, and smooth bends are replaced by polygonal joints. The change in the interplanar distances between layers of nanotubes depending on their treatment temperature was estimated from the HR TEM images. The analysis was performed using ImageJ software [48] by determining the radial profile of the FFT representation obtained from the HR TEM image of MWCNTs. It can be seen that, with an increase in the treatment temperature, the most common distance between nanotube layers shifts from 0.340 nm for the initial nanotube to 0.353 nm for nanotubes heated to 2600 °C. In addition, the intermediate sample treated at 2000 °C is characterized by the presence of two maxima at 0.334 and 0.355 nm, as well as a relatively large fraction of distances in the range of 0.4–0.55 nm, which indicates the rearrangement of the nanotube structure into a less defective one during high-temperature treatments.

#### 3.4.2. Raman Spectroscopy Characterization 

Raman spectroscopy characterization of the original and heated MWCNT samples and spectrum fitting using the 7-peak model are shown in Figure 7f. It can be seen that with an increasing treatment temperature, the contribution of all the modes associated with defective *sp^2^* carbon decreases: D_2_ (D), D_3_′ and D_3_″ and D_1_′ and D_1_″, while the fraction of G mode increases and reaches 60% at a treatment temperature of 2600 °C. This indicates a decrease in the defectiveness of nanotubes. It was found that the fraction of D_2_ (D) mode decreases with temperature from 38 to 17%, the fraction of D_3_′ mode decreases from 15 to 5%, and the fraction of D_3_″ mode decreases from 9 to 7%. A decrease in the D_3_′/D_3_″ ratio from 1.6 to 0.6 also indicates the disappearance of the main amount of graphene-like carbon fragments smaller than 1.7 nm, apparently due to rearrangement into larger fragments.

A joint analysis of TEM and Raman data allows us to assume the following changes in the structure of MWCNTs during a heat treatment. Initially, the MWCNT surface contains a larger number of graphene fragments of *sp^2^* carbon smaller than 1.7 nm (manifested as the D_3_′ mode) and larger than 1.7 nm (manifested as the D_3_″ mode). In addition, the graphene blocks that form the walls of MWCNTs are also defective (a large contribution is made by D_2_ (D) mode). Heat treatment at 2000 °C leads to an increase in the size of defect-free graphene fragments in the structure of MWCNT walls (D_2_ (D) mode decreases). In this case, graphene fragments with a size of less than 1.7 nm are spent on completing the walls of MWCNTs and are enlarged (D_3_′ mode decreases). Graphene fragments larger than 1.7 nm are involved in the completion of the MWCNT walls, and D_3_″ mode also decreases. This is confirmed by HR TEM data indicating that the structure of MWCNTs becomes more ideal and the number of single *sp^2^* fragments on their surface decreases. The treatment of MWCNTs at 2600 °C enhanced these processes, which led to an even greater decrease in the contribution of D_2_ (D) mode and an increase in G. At the same time, on the surface of MWCNTs treated at 2600 °C, *sp^2^* fragments up to 1.7 nm in size are observed, which determine the presence of D_3_′ mode in the Raman spectra, as well as the curved fragments larger than 1.7 nm (D_3_″) connecting defect-free wall blocks to each other. It should also be noted that, in contrast to the Printex-XE2-B carbon black, MWCNT graphitization at a temperature of 2600 °C proceeds more completely (the fraction of G mode for nanotubes is approximately 60% versus 35% for carbon black). This indicates the unidirectional orientation of graphene blocks in MWCNTs, which does not prevent their graphitization.

Thus, the use of the 7-peak model of fitting the Raman spectra as applied to the initial and high-temperature treated MWCNTs makes it possible to more fully determine the transformations that occur in the structure of nanotubes during annealing, characterize the change in the fraction of defective carbon and predict the properties of the obtained samples. Identification of individual modes D_3_′ and D_3_″ in the Raman spectra of amorphous carbon with fragment sizes less than or greater than 1.7 nm allows for a more accurate prediction of changes in the NCM structure, as, for example, during heat treatment.

## 4. Conclusions

Thus, complex studies of changes in the structure of amorphous carbon during heat treatment with TEM, XPS and Raman spectroscopy revealed correlations between the composition of functional surface groups, the type of hybridization of near-surface carbon atoms, and the average length of *sp^2^* carbon fragments in AC. The following conclusions were made:

1. The use of the 7-peak model for fitting the Raman spectra showed its practical applicability for studying complex systems such as amorphous carbon and made it possible to distinguish between two types of amorphous carbon, which are characterized by different modes in the Raman spectra—D_3_′ and D_3_″.
2. In addition, the separation of the contributions of D_3_′ and D_3_″ amorphous carbon modes, as well as of D_1_′ and D_1_″ modes in the 7-peak model, allows for improving the accuracy of fitting the main modes G and D_2_ (D) and obtaining a satisfactory convergence between XPS and Raman spectroscopy.
3. Our previous study on the optical properties of NDs made it possible to estimate the critical size of the graphene fragments as 1.7 nm; below it appears in the Raman spectra as the D_3_′ mode, and above it as the D_3_″ mode.
4. The use of the 7-peak model for fitting the Raman spectra confirmed the obtained data on the presence of amorphous carbon by an independent TEM method in the heat-treated samples of CB Printex-XE2-B. For the heat-treated MWCNTs, the 7-peak fitting model made it possible to isolate the change in the contribution of D_3_′ and D_3_″ modes of amorphous carbon and to determine its correspondence to the structural elements (*sp^2^* fragments on the surface, wall defects and nanotube bends).
5. The approval of the 7-peak model for fitting the Raman spectra of various NCMs—AC, heated ND, CB Printex-XE2-B and MWCNT—also showed good agreement between the Raman data and data obtained with independent methods, such as TEM, XPS and XRD in the current work and the optical properties of the heat-treated NDs and simulation of the ND graphitization process in our previous works.

Thus, the use of the 7-band model for fitting the Raman spectra of AC, ND, CB and MWCNTs demonstrated its validity and effectiveness for studying various carbon systems and its applicability in comparative studies of other NCMs.

## Figures and Tables

**Table 1:** Estimation of the length of graphene-like layers from Raman spectroscopy data and comparison with TEM data.

Temperature, °C	L_TEM_, nm	L_T-K_, nm	L_Can_, nm
800	1.3	2.6	5.8
2000	3.6	2.8	8.4
2600	4.5	4.7	10.4

L_T-K_—Tuinstra and Koenig model [59]. L_Can_—Cançado model [20].

## Data Availability

The data that support the findings of this study are available from the corresponding author upon reasonable request.
